# Association between effector-type regulatory T cells and immune checkpoint expression on CD8^+^ T cells in malignant ascites from epithelial ovarian cancer

**DOI:** 10.1186/s12885-022-09534-z

**Published:** 2022-04-21

**Authors:** Sho Sato, Hirokazu Matsushita, Daisuke Shintani, Yukari Kobayashi, Nao Fujieda, Akira Yabuno, Tadaaki Nishikawa, Keiichi Fujiwara, Kazuhiro Kakimi, Kosei Hasegawa

**Affiliations:** 1 Department of Gynecologic Oncology, Saitama Medical University International Medical Center, 1397-1 Yamane, Hidaka, Saitama 350-1298 Japan; 2 Department of Immunotherapeutics, The University of Tokyo Hospital, 7-3-1 Hongo, Bunkyo-Ku, Tokyo, 113-8655 Japan; 3 Present Address: Aichi Cancer Center Research Institute, Aichi, Japan; 4 Cancer Immunology Data Multi-Level Integration Unit, Medical Science Innovation Hub Program, RIKEN, Tokyo, Japan

**Keywords:** Ovarian cancer, Ascites, Tregs, CCR4, PD-1, T cells

## Abstract

**Background:**

Regulatory T cells (Tregs) play an important role in the antitumor immune response in epithelial ovarian cancer (EOC). To understand the immune-inhibitory networks of EOC, we addressed the association between Tregs and immune checkpoint expression on T cells in the tumor microenvironment of EOC.

**Methods:**

A total of 41 patients with stage IIIC and IV EOC were included in the analysis. We harvested cells from malignant ascites and investigated them using multi-color flow cytometry. We categorized the Tregs into 3 groups: effector-type Tregs, naïve Tregs and non-Tregs, based on the expression patterns of CD45RA and Foxp3 in CD4^+^ T cells. Furthermore, the relationships between the expression of various immune checkpoint molecules, such as PD-1, on CD8^+^ T cells and each of the Treg subtypes was also evaluated.

**Results:**

The median frequency of naïve Tregs, effector-type Tregs and non-Tregs were 0.2% (0–0.8), 2.0% (0–11.4) and 1.5% (0.1–6.3) in CD4^+^ T cells of malignant ascites from EOC patients, respectively. A high frequency of effector-type Tregs was associated with high-grade serous carcinoma compared with the other histotypes. Patients with higher proportions of effector-type Tregs showed a trend towards increased progression-free survival. We also demonstrated a correlation between a higher proportion of effector-type Tregs and increased PD-1 expression on CD8^+^ T cells. In addition, C–C chemokine receptor 4 expression was also observed in effector-type Tregs.

**Conclusion:**

These data suggest that multiple immune-inhibitory networks exist in malignant ascites from EOC patients, suggesting an approach towards combinational immunotherapies for advanced EOC patients.

**Supplementary Information:**

The online version contains supplementary material available at 10.1186/s12885-022-09534-z.

## Background

Epithelial ovarian cancer (EOC) is the fifth leading cause of cancer deaths among women in the United States and the mortality of EOC is the highest of all gynecologic cancers [1]. It is generally characterized by few early symptoms, widespread peritoneal dissemination, and ascites in the advanced stage.

The majority of EOC cases (60%) are diagnosed in the advanced stage, and its mean 5-year survival rate is 29% [2]. The standard treatment for EOC is cytoreductive surgery and combination chemotherapy with carboplatin and paclitaxel. In general, patients respond very well to this protocol. However, most of the patients with advanced EOC experience relapse or develop metastatic disease. The peritoneal cavity is the most frequent site of recurrence for EOC patients, and most patients eventually become chemo-resistant and die from their disease [3].

In recent years, regulatory T cells (Tregs) have received an attention in the tumor immunosuppressive environment. Tregs play an indispensable role in maintaining immunological hyporesponsiveness to self-antigens and in suppressing excessive immune responses that would be deleterious to the host in healthy humans [4]. Tregs are produced in the thymus, as a functionally mature subpopulation of T cells, and can also be induced from naïve T cells in the periphery [4]. On the other hand, there is evidence supporting the contribution of Tregs to immune dysfunction in cancer patients [5]. FoxP3 is a key regulatory gene for Tregs [6]. Recently, it has become clear that human FoxP3^+^ CD4^+^ T cells are comprised of three functionally and phenotypically distinct subpopulations. CD45RA^+^ FoxP3^lo^ Treg cells (naïve Tregs), CD45RA^−^ FoxP3^hi^ Treg cells (effector-type Tregs), both of which are suppressive in vitro, and cytokine-secreting CD45RA^−^ FoxP3^lo^ non-suppressive T cells (non-Tregs) [7]. The majority of cancers are infiltrated predominantly by effector-type Tregs [8]. Nakayama et al. reported that effector-type Tregs are associated with worse prognosis in patients with diffuse large B-cell lymphoma [9]. Lin et al. indicated that effector-type Tregs were associated with tumor metastasis in colorectal cancer [10]. However, these Treg subtypes have not yet been investigated in patients with EOC.

C–C chemokine receptor 4 (CCR4) is important for regulating immune balance and is known to be expressed selectively on Th2 cells and Tregs [11]. Sugiyama et al. found that CCR4 was specifically expressed by a subset of terminally differentiated and most suppressive CD45RA^−^ FOXP3^hi^ CD4^+^ Tregs, which is designated effector type Tregs, in tumors and peripheral blood. CCR4^+^ effector-type Tregs are the predominant phenotype among tumor-infiltrating FoxP3^hi^ CD4^+^ T cells and are much higher in tissues compared with peripheral blood in patients with melanoma [12]. Anti-CCR4 monoclonal antibody (Mogamulizumab) has been used to treat Adult T-cell leukemia-lymphoma (ATL) patients. Almost all ATL cells express CCR4 and are thus the direct target of antibody-mediated depletion [13]. However, CCR4 expression on effector-type Tregs in EOC has not been investigated to date.

Recent clinical trials of PD-1 blockade therapy for EOC have led to unsatisfactory results [14]. It is possible that multiple immune-inhibitory mechanisms exist in EOC. To address this question, malignant ascites represents an ideal source for investigating the tumor-immune microenvironment because the cells essentially exist in a suspension. Therefore, it is easy to assess both immune and tumor cells by flow cytometric analysis simultaneously. We previously observed multiple immune checkpoint molecules on T cells in the tumor microenvironment of EOC through analysis of ascites cells [15].

In this study, we focused on Tregs and the subtypes in malignant ascites from EOC patients and investigated their clinical significance. Furthermore, the expression of immune checkpoint molecules on CD8^+^ T cells, such as PD-1, TIM-3, CTLA4 and BTLA, was also determined to better understand the multiple immune-inhibitory networks in advanced EOC.

## Methods

### Patients

This study was reviewed and approved by the Institutional Review Board of Saitama Medical University International Medical Center (No.13–092). A total of Forty-one patients who were diagnosed as having advanced EOC (FIGO stage IIIC and IV), had malignant ascites and were treated at Saitama Medical University International Medical Center between December 2010 and November 2014, were included in this study. All patients were chemo-naïve. The median age of the patients was 65 years with a range of 41–85 years. The EOC cases consisted of 31 (75.6%) stage IIIC and 10 (24.4%) stage IV patients. There were 25 (61.0%) serous, 7 (17.1%) clear cell and 9 (21.9%) other types of carcinomas. (Table 1).

**Table 1:** Association between effector-type Tregs and clinicopathological features in advanced EOC

Factors	High effector-type Tregs/ Cases (%)	*P* value
Age		
< 65	10/19 (52.6)	0.43
65 ≤	11/22 (50.0)	
Histology		
High-grade serous	16/25 (64.0)	0.042^a^
Clear cell	2/7 (28.6)	
Others	3/9 (50.0)	
Stage		
IIIC	15/31 (48.4)	0.71
IV	6/10 (60.0)	
Tumor size		
< 100 mm	14/22 (63.6)	0.13
100 mm ≤	7/19 (36.8)	
Residual tumor		
Yes	20/38 (52.6)	0.85
No	1/3 (33.3)	

^a^ High-grade serous vs. Clear cell and Others

### Ascites cells and flow cytometry

Ascites was collected before the patients underwent their initial treatment, either primary debulking surgery or neoadjuvant chemotherapy. The harvested cells were stored in cell freezing medium at -80 °C until analysis.

The following monoclonal antibodies (mAbs) were used for flow cytometry: FITC-labeled anti-human CD4 (BD Biosciences, San Diego, CA) and mouse IgG_1_ isotype (BioLegend, San Diego, CA) antibodies, PE-labeled anti-human CD279 (PD-1) (BioLegend), anti-human CD366 (TIM-3) (BioLegend), anti-human CD272 (BTLA) (BioLegend), anti-human LAG3 (R&D Systems Inc., Minneapolis, MN), anti-human CD25 (BioLegend) and mouse IgG_1_ isotype (BioLegend) antibodies, PC5-labeled anti-CD3 (BioLegend) and anti-CD194 (CCR4) (BioLegend) antibodies, APC-labeled anti-CD45 (BioLegend) and mouse IgG_1_ isotype (BioLegend) antibodies, Pacific Blue-labeled anti-CD8a (BioLegend) and anti-CD4 (BioLegend) antibodies, and ECD-labeled CD45RA antibody (Beckman Coulter, San Diego, CA). Fixable Viability Dye eFluor 780 (eBioscience, San Diego, CA) was used to exclude dead cells. Ascites cells were harvested by centrifugation, stained with the mAbs described above. Intracellular staining for Foxp3 was performed using a FITC-labeled anti-human Foxp3 antibody (BioLegend) and FOXP3 Fix/Perm Buffer Set (BioLegend) according to the manufacturer's instructions. The cells were then analyzed on a Gallios flow cytometer (Beckman Coulter). The data were processed using Kaluza software (Beckman Coulter).

### Statistical analysis

Differences between the groups of patients were assessed by one-way ANOVA, Student’s t-test, or Chi-square test. Survival was assessed using the Kaplan–Meier estimator method, and any significant differences between groups were determined using the log-rank test. Statistical analyses were performed using GraphPad Prism 6.0 (GraphPad Software, San Diego, CA). All reported p-values were two-sided, and a value of *p* < 0.05 was considered significant.

## Results

### Tregs in malignant ascites from advanced EOC patients

Regulatory T cells (Tregs) have been reported to play an important role in the immune inhibitory network of EOC [16]. Subtype-specific biology of Tregs has been observed in some types of cancer but not in EOC [17]. Therefore, we investigated the prevalence of Tregs and the subtypes, based on the expression patterns of FoxP3 and CD45RA on CD4^+^ T cells, in malignant ascites from 41patients with advanced EOC. Gating strategy for Tregs was shown in Supplementary Fig. 1.

**Fig. 1:**
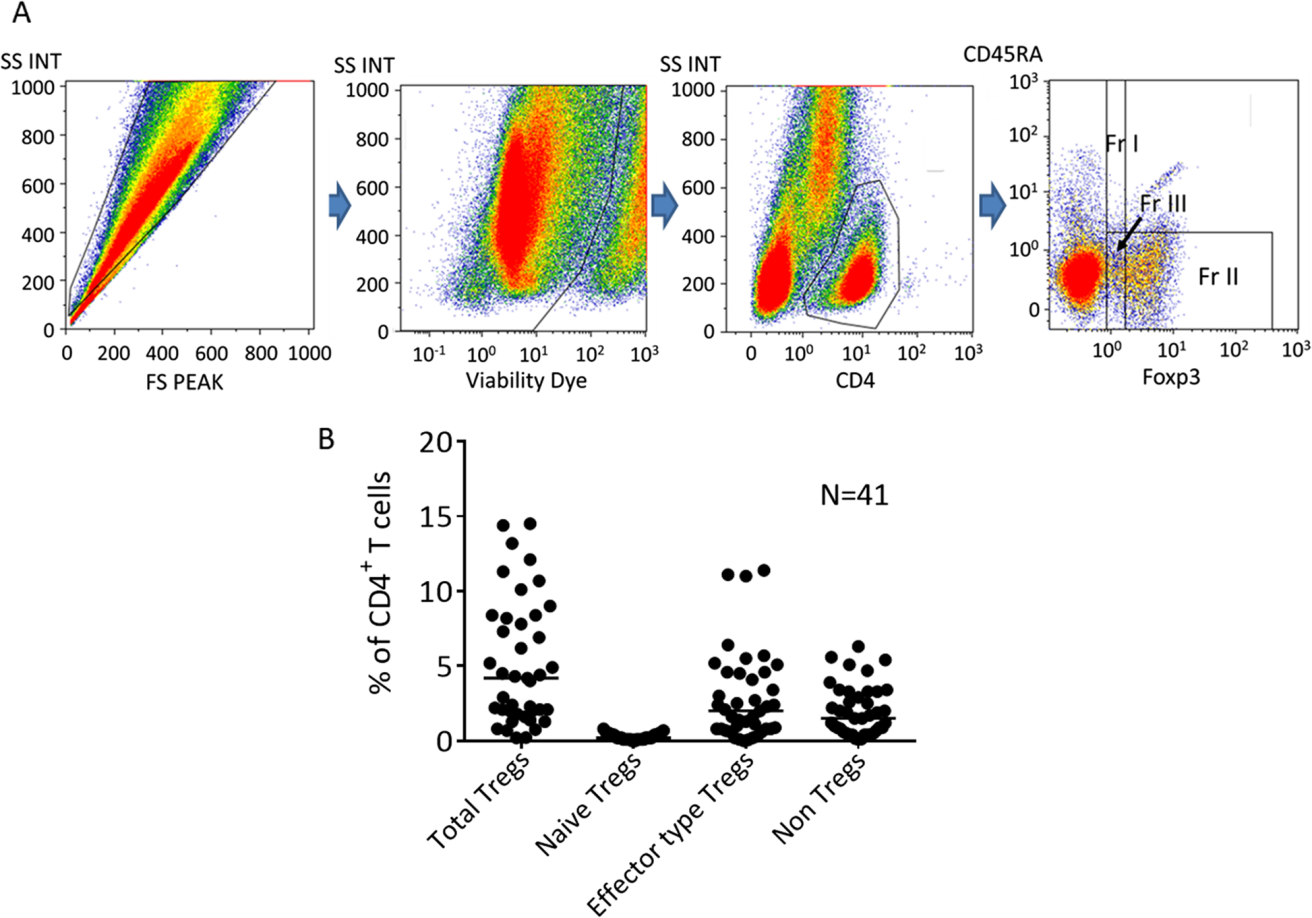
**A** Analysis of Tregs and subtypes in malignant ascites from EOC by multicolor flow cytometry. Fr I: naïve Tregs, Fr II: effector-type Tregs, Fr III: non-Tregs. FS, forward scatter; SS, side scatter; INT, integral. **B** Frequency of total Tregs and subtypes; naïve Tregs, effector-type Tregs and non-Tregs in malignant ascites from EOC. Bars indicate the median value (%)

Figure 1A shows the representative analysis pipeline for Tregs in ascites cells from advanced EOC. We examined the frequency of Tregs and their subtypes (CD45RA^+^ FoxP3^lo^, naïve Tregs, CD45RA^−^ FoxP3^hi^ effector-type Tregs, and CD45RA^−^ FoxP3^lo^ as non-Tregs) among all CD4^+^ T cells in the ascites from each patient. The median value of frequency of CD4^+^ T cells was 30.5% (range:10.4–71.9) of CD3^+^ T cells. As illustrated in Fig. 1B, Tregs comprised 4.2% (range,0.2–14.5%) of all CD4^+^ T cells in ascites, and 0.2% (0–0.8%), 2.0% (0–11.4%) and 1.5% (0.1–6.3%) of CD4^+^ T cells were naïve Tregs, effector-type Tregs and non-Tregs, respectively.

### Relationship between effector-type Tregs and clinicopathological factors in patients with advanced EOC

Recent reports have described the importance in evaluating effector-type Tregs rather than total Tregs [12, 17]. We queried whether there were any correlations between effector-type Tregs and clinical factors. Table 1 summarizes the relationship between clinicopathological features and the proportion of effector-type Tregs among all CD4^+^ T cells in the ascites. We defined the patients who exhibited high or low frequency of effector-type Tregs based on the median value (2.0%). The effector-type Tregs were observed significantly higher frequency in patients with high-grade serous carcinoma (HGSC) compared with those in non-serous histological types (*P* = 0.042, Student's t-test). No significant correlation was found between the patients with high frequency of effector-type Tregs and other clinicopathological variables.

### Effector-type Tregs and outcomes in advanced EOC patients

**Fig. 2:**
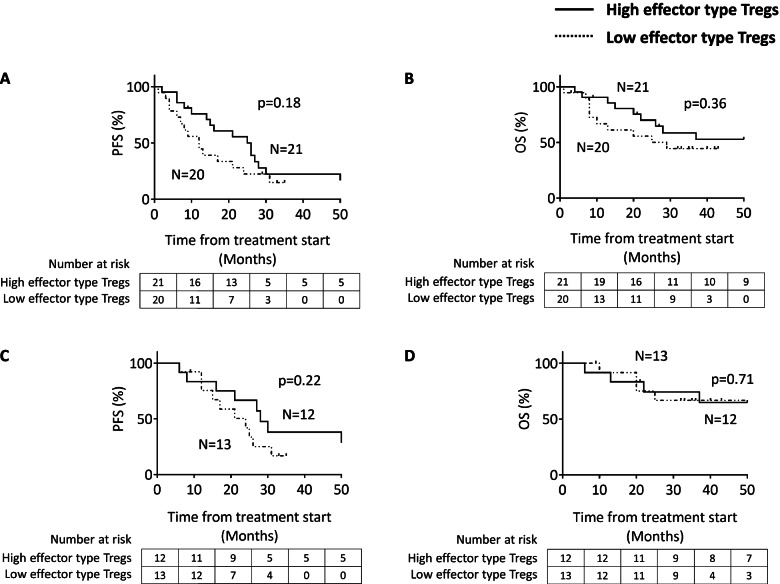
Kaplan–Meier curves for progression free survival (PFS) and overall survival (OS) stratified by the median value of the frequency of effector-type Tregs from all CD4^+^ T cells. A and B in all patients. C and D in patients with HGSC

We analyzed the potential association between the effector-type Tregs and outcomes in advanced EOC patients. Of the 41 patients included in the analysis, the median follow-up for all patients was 20 months. Based on the median value of effector-type Tregs among all CD4^+^ T cells, the patients with higher frequency for effector-type Tregs showed a trend for increased progression-free survival (PFS). However, there was survival difference in neither PFS (*p* = 0.18; Fig. 2A) nor overall survival (OS) (*p* = 0.36; Fig. 2B) in all EOC patients. Next, we analyzed in a subgroup of patients with HGSC because they had a higher frequency of effector-type Tregs than other histotypes (Table 1). No correlation was observed between the outcomes and effector-type Tregs in patients with HGSC, but with a trend toward better PFS for effector-type Tregs. (Fig. 2C and D).

#### CCR4 expression on effector-type Tregs from advanced EOC patients

**Fig. 3:**
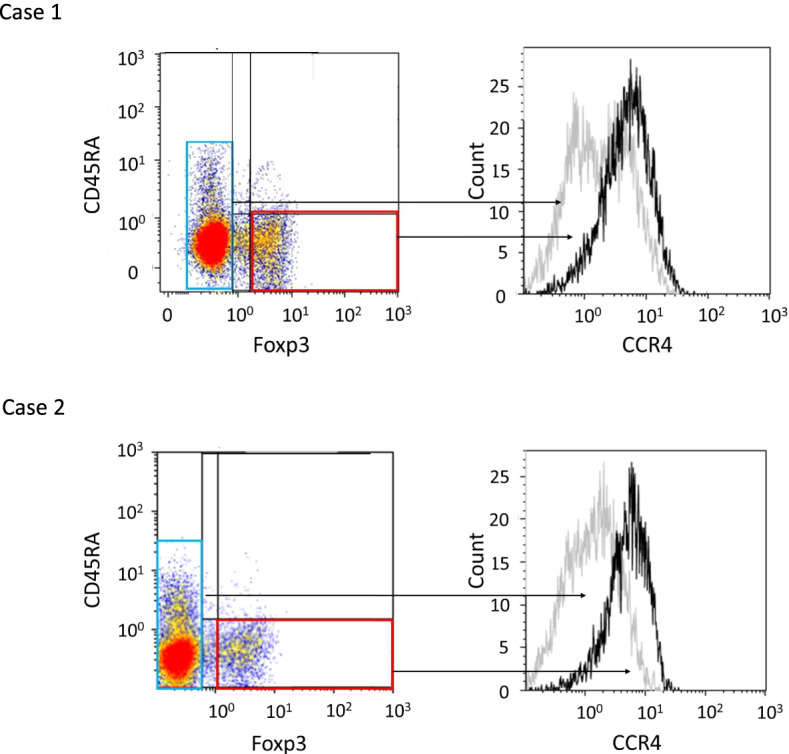
C–C chemokine receptor 4 (CCR4) expression in subpopulations based upon the FOXP3 expression status in ascites from EOC patients. CCR4 expression was evaluated for CD45RA^−^ FOXP3^hi^ (effector-type Tregs). Box 1 and 2 represent FOXP3^−^ cells and CD45RA^−^ FOXP3^hi^ cells among all CD4^+^ T cells, respectively. Representative cases are shown

C–C chemokine receptor 4 (CCR4) has been reported to be predominantly expressed on the cell surface of effector-type Tregs, in both cancer tissues and peripheral blood from patients [12]. We investigated whether effector-type Tregs in malignant ascites from advanced EOC patients express CCR4. CCR4 expression was observed in effector-type Tregs (Fig. 3 and Supplementary Fig. 2).

#### Association between the expression of immune checkpoint molecules on CD8^+^ T cells and effector-type Tregs in ascites

To elucidate the multiple immune-inhibitory networks in malignant ascites of EOC, we investigated the association between the expression of immune checkpoint molecules on CD8^+^ T cells and the frequency of Tregs in ascites. The median value of frequency of CD8^+^ T cells was 57.4% (range:24.6–87.8) of CD3^+^ T cells. Gating strategy for an inhibitory molecule on CD8^+^ T cells was shown in Supplementary Fig. 3.

**Fig. 4:**
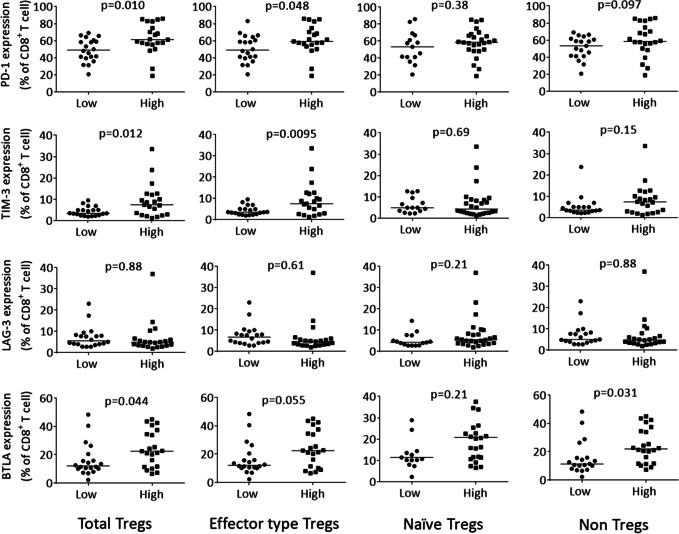
Expression rates of PD-1, TIM-3, LAG-3 and BTLA on CD8^+^ T cells in patients with either high or low frequency of Treg subtypes. Bars indicate median value (%) of each immune checkpoint expression rate

As shown in Fig. 4, the expression of PD-1 (*p* = 0.048) and TIM-3 on CD8^+^ T cells (*p* = 0.0095) were significantly greater in patients with higher frequency of effector-type Tregs than in those with fewer effector-type Tregs, based on the median value. We next investigated the associations between the expression of each of the four immune checkpoint molecules (PD-1, TIM-3, LAG-3 and BTLA) and the frequency of effector-type Tregs (Supplementary Table 1). We observed that 57.1% of the advanced EOC patients exhibiting high PD-1 expression on the CD8^+^ T cells also showed a high frequency of effector-type Tregs in ascites, suggesting multiple immune-inhibitory mechanism in malignant ascites of advance EOC patients.

## Discussion

In this study, we investigated the frequency of Tregs and their subtypes among CD4^+^ T cells in ascites from advanced EOC patients. We found that HGSC patients exhibited higher proportions of effector-type Tregs than those with other histotypes. We also demonstrated an association between the higher frequency of effector-type Tregs and high PD-1 expression on CD8^+^ T cells in ascites from advanced EOC. Additionally, CCR4 expression was observed in effector-type Tregs in ascites from advanced EOC.

Miyara et al. reported that Tregs in humans were heterogeneous in phenotype and function, consisting of suppressive and non-suppressive subpopulations, and could be categorized into three subtypes naïve Tregs, effector-type Tregs and non-Tregs [7].

Nishikawa et al. reported the importance of addressing effector-type Tregs because of their biological and clinical features [18]. This is the first report to investigate effector-type Tregs in EOC patients. Our study did not indicate any differences in survival between the high and low frequency of effector-types Tregs, although a trend toward improved survival among high effector-type Tregs was observed. Curiel et al. analyzed ascites from 45 and tissues from 104 untreated EOC patients and reported that patients with Tregs in their tumors were associated with high death hazard and reduced survival [16]. In contrast, some reports have described high infiltration of Tregs in EOC were associated with better prognosis. Milne et al. investigated tissue microarrays of 199 HGSC from optimally debulked patients and found that the presence of intraepithelial FoxP3^+^ cells was associated with increased disease-specific survival [19]. Tsiatas et al. reported that cells from 45 tumors from EOC patients were analyzed by flow cytometry, and patients with a higher number of CD4^+^ CD25^hi^ cells had significantly improved PFS and OS [20]. A recent review summarized associations between Tregs and survival in EOC and concluded that the reason why Tregs had different impact on survivals as described above remained unclear [21].

We demonstrated that the high proportion of effector-type Tregs associated with high levels of expression of PD-1 and TIM-3 on CD8^+^ T cells, suggesting multiple immune-inhibitory networks in the tumor microenvironment of EOC. These results are concordant with a previous report in hepatocellular carcinoma (HCC). Kalathil et al. performed a global analysis of immune dysfunction in HCC and reported that HCC exploited multiple immunosuppressive mechanisms to evade active immune surveillance of the host, such as induction of Tregs, recruitment of Myeloid -derived suppressor cells, expression of PD-1 on T cells and increased production of inhibitory cytokines [22, 23].

Early phase studies of PD-1/L1 blockade therapy for EOC have reported response rates below 15% [24–26]. A recent, large, phase 2 study of Keynote 100 for recurrent EOC patients, who had already undergone treatment with platinum-based therapy, showed a response rate of only 8% [27]. Although some of the EOCs were reported to be “immune hot tumors” [28, 29], the efficacy of PD-1/L1 blockade therapy was not as high as was expected. Our results indicated that about 60% of patients exhibiting high PD-1 expression on CD8^+^ T cells also exhibited a high frequency of effector-type Tregs. This can partly explain the low response rates of single PD-1/L1 blockade therapy in EOC patients. Targeting both Tregs and PD-1/L1 pathways might provide a clue to the development of an efficient immunotherapy for EOC.

Effector-type Tregs can be eliminated through administration of an anti-CCR4 monoclonal antibody, Mogamulizumab, which was originally developed for the treatment of ATL patients [12]. A phase Ia clinical trial of Tregs depletion by the administration of Mogamulizumab for advanced or recurrent solid tumor patients revealed a significant reduction of effector type Tregs in the peripheral blood [30].　The combination therapy of Nivolumab and Mogamulizumab provided an acceptable safety profile, antitumor activity for solid tumors which were including esophageal cancer, gastric cancer, pancreatic cancer, and small-cell lung cancer in phase I study [31]. Our results support the evaluation of this combination therapy in EOC patients.

Our study has several limitations. One is the retrospective design with the previously collected samples. The second is that all samples were collected and stored at -80 ℃ before analysis. We do not have the answer if this had any effect on the results. The third we evaluated each cell type by the rate of cells only. We could not analyze each cell type by total number of the cells because the data about total amount of ascites was unavailable.

## Conclusions

In conclusion, we reported effector-type Tregs in EOC for the first time and demonstrated that multiple immune-inhibitory networks existed in the tumor microenvironment of EOC. A combinational immunotherapy targeting both effector-type Tregs and immune checkpoints axes, may overcome the limited efficacy of immunotherapy for EOC to date.

## Supplementary Information

**Additional file 1.**

**Additional file 2.**

**Additional file 3.**

**Additional file 4.**

## Data Availability

The datasets used and/or analysed during the current study available from the corresponding author on reasonable request.

## Abbreviations

Tregs: Regulatory T cells
CCR4: C–C chemokine receptor 4
PD-1: Programmed cell death-1
LAG-3: Lymphocyte-Activation Gene-3
TIM-3: T-cell immunoglobulin and mucin-domain containing-3
BTLA: B And T Lymphocyte Attenuator
EOC: Epithelial ovarian cancer
HGSC: High grade serous carcinoma
PFS: Progression free survival
OS: Overall survival
