# Supplementary material for: Association between effector-type regulatory T cells and immune checkpoint expression on CD8+ T cells in malignant ascites from epithelial ovarian cancer
Source: BMC Cancer. 2022 Apr 21;22:437. doi: 10.1186/s12885-022-09534-z (PMC9026673; doi:10.1186/s12885-022-09534-z)
Supplement: Supplementary file 1 — Additional file 1. [file 12885_2022_9534_MOESM1_ESM.pptx]

## Slide 1
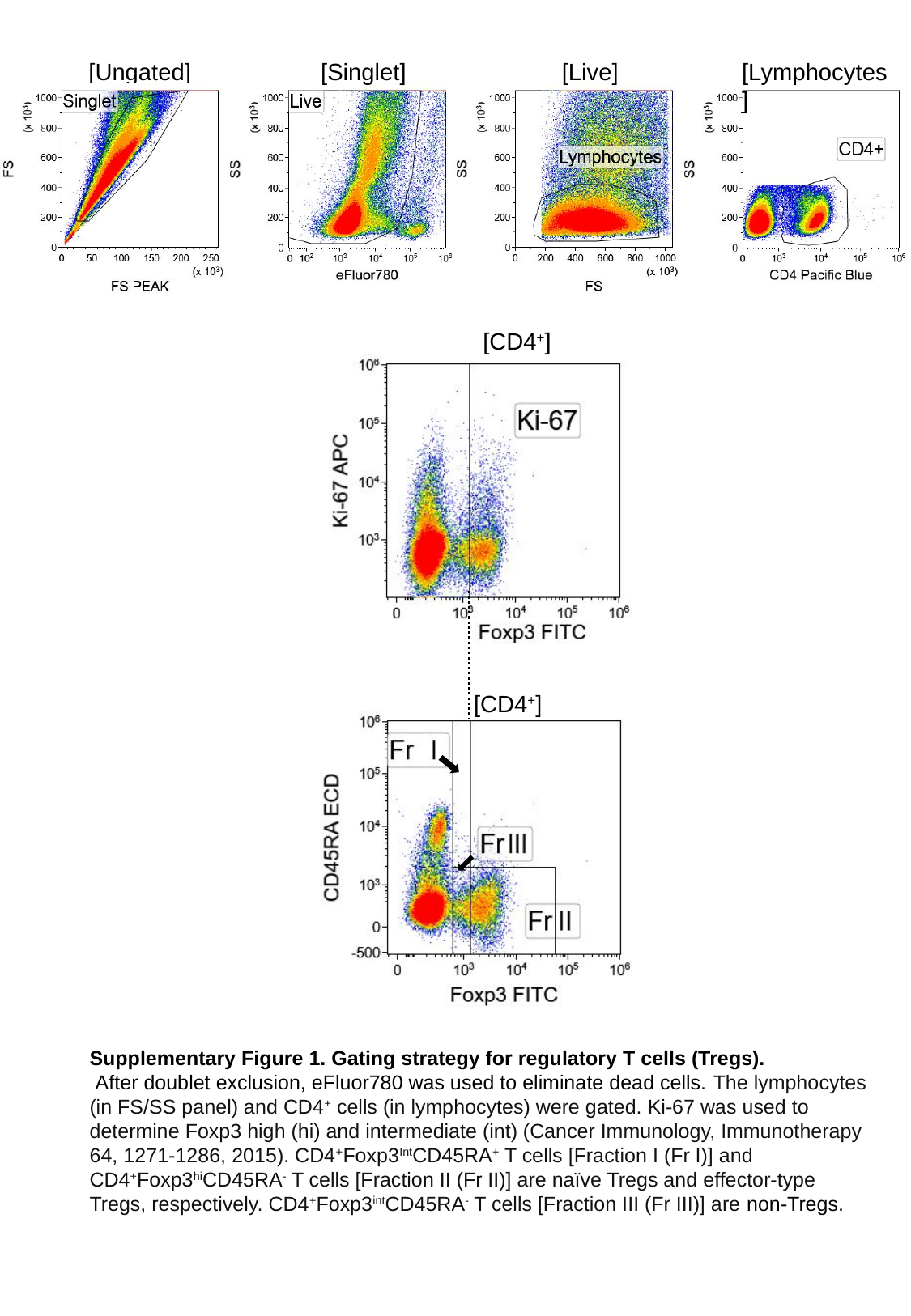

[Ungated]
[Singlet]
[Live]
[Lymphocytes]
[CD4+]
[CD4+]
Supplementary Figure 1. Gating strategy for regulatory T cells (Tregs).
 After doublet exclusion, eFluor780 was used to eliminate dead cells. The lymphocytes (in FS/SS panel) and CD4+ cells (in lymphocytes) were gated. Ki-67 was used to determine Foxp3 high (hi) and intermediate (int) (Cancer Immunology, Immunotherapy 64, 1271-1286, 2015). CD4+Foxp3IntCD45RA+ T cells [Fraction I (Fr I)] and CD4+Foxp3hiCD45RA- T cells [Fraction II (Fr II)] are naïve Tregs and effector-type Tregs, respectively. CD4+Foxp3intCD45RA- T cells [Fraction III (Fr III)] are non-Tregs.
