# Supplementary material for: Association between effector-type regulatory T cells and immune checkpoint expression on CD8+ T cells in malignant ascites from epithelial ovarian cancer
Source: BMC Cancer. 2022 Apr 21;22:437. doi: 10.1186/s12885-022-09534-z (PMC9026673; doi:10.1186/s12885-022-09534-z)
Supplement: Supplementary file 2 — Additional file 2. [file 12885_2022_9534_MOESM2_ESM.pptx]

## Slide 1
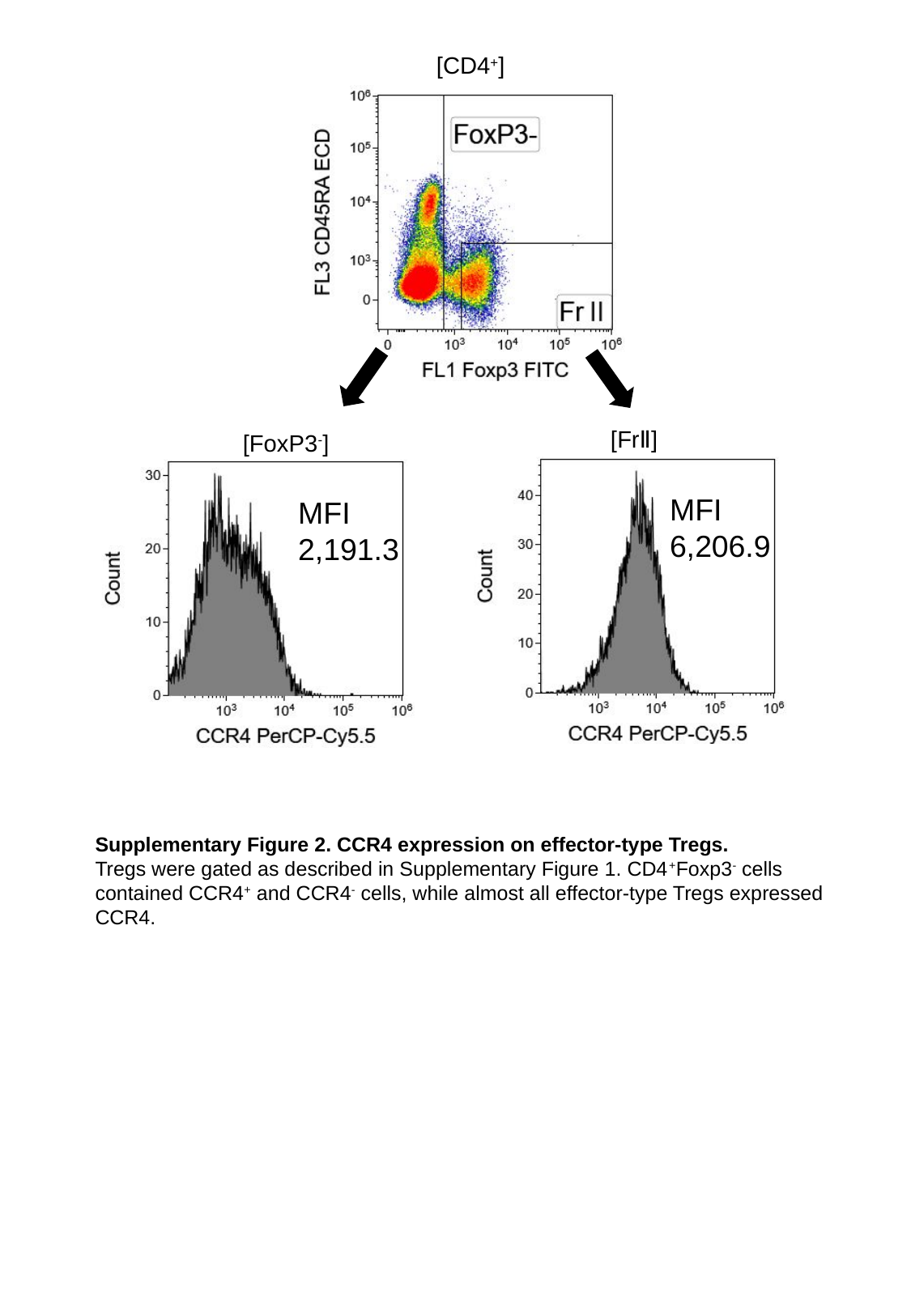

[CD4+]
[FrⅡ]
[FoxP3-]
MFI
6,206.9
MFI
2,191.3
Supplementary Figure 2. CCR4 expression on effector-type Tregs.
Tregs were gated as described in Supplementary Figure 1. CD4+Foxp3- cells contained CCR4+ and CCR4- cells, while almost all effector-type Tregs expressed CCR4.
