# Supplementary material for: Association between effector-type regulatory T cells and immune checkpoint expression on CD8+ T cells in malignant ascites from epithelial ovarian cancer
Source: BMC Cancer. 2022 Apr 21;22:437. doi: 10.1186/s12885-022-09534-z (PMC9026673; doi:10.1186/s12885-022-09534-z)
Supplement: Supplementary file 3 — Additional file 3. [file 12885_2022_9534_MOESM3_ESM.pptx]

## Slide 1
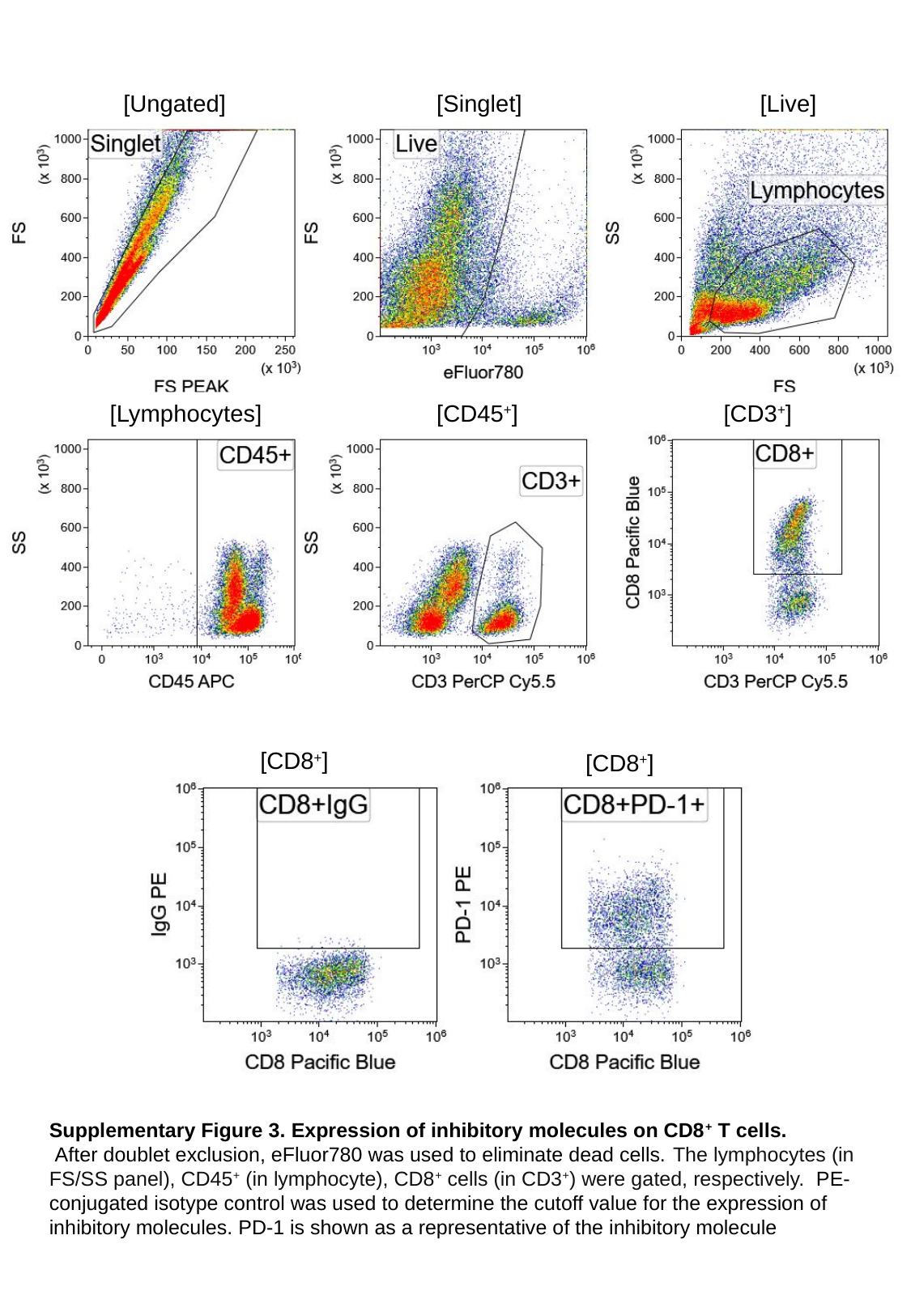

[Ungated]
[Singlet]
[Live]
[CD3+]
[CD45+]
[Lymphocytes]
[CD8+]
[CD8+]
Supplementary Figure 3. Expression of inhibitory molecules on CD8+ T cells.
 After doublet exclusion, eFluor780 was used to eliminate dead cells. The lymphocytes (in FS/SS panel), CD45+ (in lymphocyte), CD8+ cells (in CD3+) were gated, respectively. PE-conjugated isotype control was used to determine the cutoff value for the expression of inhibitory molecules. PD-1 is shown as a representative of the inhibitory molecule
