# Supplementary material for: Association between effector-type regulatory T cells and immune checkpoint expression on CD8+ T cells in malignant ascites from epithelial ovarian cancer
Source: BMC Cancer. 2022 Apr 21;22:437. doi: 10.1186/s12885-022-09534-z (PMC9026673; doi:10.1186/s12885-022-09534-z)
Supplement: Supplementary file 4 — Additional file 4. [file 12885_2022_9534_MOESM4_ESM.docx]

**Supplementary Table 1. Associations between Effector type Tregs and expression of immune checkpoint molecules on CD8^+^ T cells**

| Immune check point  molecules on CD8^+^ T cells | High effector-type Tregs / Cases (%) |
| --- | --- |
| PD-1 |  |
| High | 12/21 (57.1%) |
| Low | 9/20 (45.0%) |
| TIM-3 |  |
| High | 9/21 (42.9%) |
| Low | 12/20 (60.0%) |
| LAG-3 |  |
| High | 15/22 (68.2%) |
| Low | 6/19 (31.6%) |
| BTLA |  |
| High | 15/21 (71.4%) |
| Low | 6/20 (30.0%) |
